# Exocytosis and Endocytosis: Yin-Yang Crosstalk for Sculpting a Dynamic Growing Pollen Tube Tip

**DOI:** 10.3389/fpls.2020.572848

**Published:** 2020-10-06

**Authors:** Lifeng Zhao, Muhammad Saad Rehmani, Hao Wang

**Affiliations:** College of Life Sciences, South China Agricultural University, Guangzhou, China

**Keywords:** endocytosis, exocytosis, pollen tube growth, cell polarity, mathematical modeling

## Abstract

The growing pollen tube has become one of the most fascinating model cell systems for investigations into cell polarity and polar cell growth in plants. Rapidly growing pollen tubes achieve tip-focused cell expansion by vigorous anterograde exocytosis, through which various newly synthesized macromolecules are directionally transported and deposited at the cell apex. Meanwhile, active retrograde endocytosis counter balances the exocytosis at the tip which is believed to recycle the excessive exocytic components for multiple rounds of secretion. Therefore, apical exocytosis and endocytosis are the frontline cellular processes which drive the polar growth of pollen tubes, although they represent opposite vesicular trafficking events with distinct underpinning mechanisms. Nevertheless, the molecular basis governing the spatiotemporal crosstalk and counterbalance of exocytosis and endocytosis during pollen tube polarization and growth remains elusive. Here we discuss recent insight into exocytosis and endocytosis in sculpturing high rates of polarized pollen tube growth. In addition, we especially introduce the novel integration of mathematical modeling in uncovering the mysteries of cell polarity and polar cell growth.

## Introduction

Cell polarity and polar cell growth play essential roles in a wide range of biological processes by regulating cell growth, development, patterning, communication and signaling (Campanale et al., 2017; Muroyama and Bergmann, 2019). The growing pollen tube is regarded as one of the ideal cell model systems, similar to budding yeasts and neuron synapses, to study cell polarity and polar cell growth in plants (Hepler et al., 2001; Winship et al., 2011; Cai and Del Duca, 2019). Pollen tube growth is featured by its polarized and rapid tip expansion. It can reach up to 2.8 μm s^–1^ in maize and 0.2–0.3 μm s^–1^ in lily (Hepler et al., 2001; Stone et al., 2004). Moreover, the molecular mechanisms of pollen tube guidance and navigation have been well documented in previous studies (Grebnev et al., 2017; Cameron and Geitmann, 2018; Zhong and Qu, 2019; Duan et al., 2020). However, a key unanswered question is how polarization and polar growth of the pollen tubes are intracellular empowered and maintained.

**FIGURE 1:**
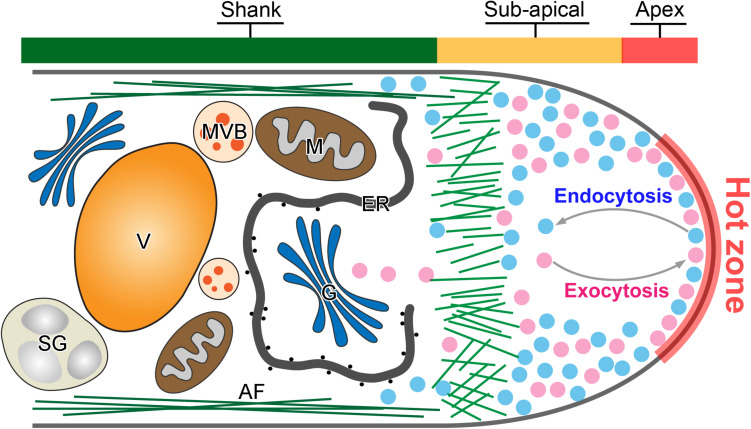
Tip-focused exocytosis and endocytosis in the pollen tube tip. A representative demonstration of the intracellular structure of a pollen tube tip. Active exocytosis and endocytosis occur simultaneously in the hot zone of pollen tube tip to drive the rapid and polarized pollen tube growth. Large amounts of exocytic (purple) and endocytic (blue) vesicles are accumulated in the pollen tube tip. In contrast, large organelles such as Golgi stacks (G), endoplasmic reticulum (ER) and mitochondria (M), multivesicular body (MVB), starch granule (SG), and vacuole (V) move along actin filament (AF) in the pollen tube shank.

Anterograde exocytosis mediates the vesicle secretion from pollen tube shank to the apical region for fusion. It functions as the front line of intracellular activity that contributes directly to the pollen tube tip expansion (Figure 1). Newly synthesized macromolecules including proteins, lipids and cell wall materials are packed into exocytic vesicles and are transported from the pollen tube shank to the tip region (McKenna et al., 2009; Cameron and Geitmann, 2018; Meng et al., 2020). The vesicles fuse then with the apical plasma membrane (PM) and discharge their internal cargoes to provide the new materials for the fast expansion of the pollen tube apex (Bloch et al., 2016; Luo et al., 2017). However, earlier morphometric analyses of vesicle secretion at the pollen tube tip revealed that more exocytic vesicles fuse with the PM than are required to satisfy the demands of surface expansion (Derksen et al., 1995; Ketelaar et al., 2008). Therefore, retrograde endocytosis which is the opposite vesicle trafficking in the pollen tube tip, counteracts with anterograde exocytosis to maintain a dynamic balance in the apical dome (Figure 1). It takes place simultaneously to recycle the excessive fusion-unsuccessful exocytic vesicles for multiple rounds of secretion and fusion, and internalize vesicles invaginated from the apical PM (Wang et al., 2005; Zonia and Munnik, 2008; Grebnev et al., 2017; Kaneda et al., 2019). It functions in pollen tube guidance, signal transduction and nutrient uptake (Sekeres et al., 2015; Hao et al., 2016; Hempel et al., 2017; Wang et al., 2020). Together, polarization and polar growth of pollen tubes are empowered by both exocytosis and endocytosis which are two antagonistic intracellular processes as shown in Figure 1.

In addition to exocytosis and endocytosis, an intricate regulatory network consisting of plant-specific Rho GTPase 1 (ROP1), actin cytoskeleton, exocyst complex proteins, phospholipids (PIs), SNARE proteins, cell wall biochemical mechanics, Ca^2+^, pH, reactive oxygen species (ROS) and so forth regulates exocytosis and endocytosis in the pollen tube tip (Lee et al., 2008; Craddock et al., 2012; Duan et al., 2014; Zhou et al., 2015; Takeuchi and Higashiyama, 2016; Sekeres et al., 2017; Mangano et al., 2018; Wudick et al., 2018; Do et al., 2019; Domingos et al., 2019; Guo and Yang, 2020). Nevertheless, little is known about how these different regulatory factors are well orchestrated to function as positive and negative feedback loops in their action on exocytosis and endocytosis and thereby governing pollen tube polarization and polar growth.

In this mini review, due to space constrains we predominantly focus on the recent insights into exocytosis and endocytosis in regulating cell polarization and polar cell growth. In addition, we discuss the novel integration of mathematical modeling in underlying the roles of exocytosis and endocytosis in cell polarization and growth, and highlight a few outstanding questions that needed to be addressed to understand the mechanisms by which pollen tube polarity and growth are regulated.

## The Origin and Identity of Tip-Focused Exocytosis and Endocytosis in Growing Pollen Tubes

**FIGURE 2:**
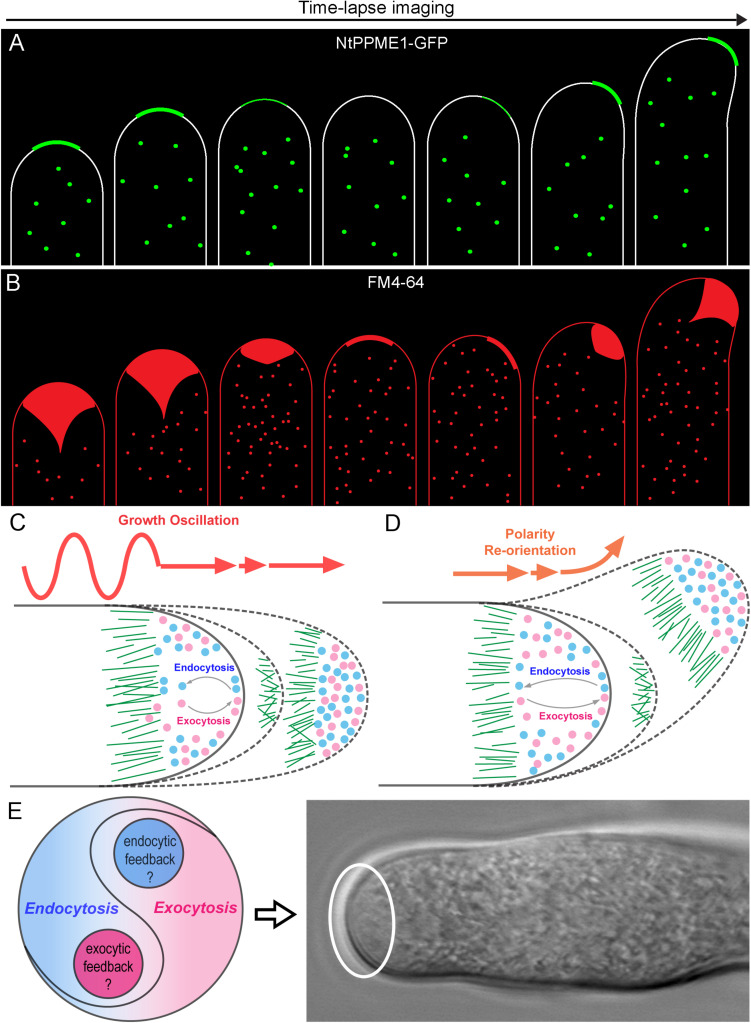
The yin-yang crosstalk of apical exocytosis and endocytosis during pollen tube tip growth oscillation and guidance. **(A)** A model of time-lapse images of apical subcellular localization of NtPPME1-GFP during with pollen tube growth oscillation and polarity shift based on the results from previous studies (Bosch et al., 2005; Wang et al., 2013, 2016). **(B)** A model of time lapse images of tip-focused endocytosis stained by FM4-64 which is derived from previous studies shows that apical endocytosis is closely associated with growth oscillation and polarity re-orientation during pollen tube growth (Zonia and Munnik, 2008; Onelli and Moscatelli, 2013). **(C,D)** Hypothetical models for illustrating the dynamic changes of the apical exocytosis and endocytosis during pollen tube growth oscillation and guidance. **(E)** A hypothetical model of the yin-yang crosstalk and counterbalance between exocytosis and endocytosis occur in the pollen tube tip highlighted by the white cycle.

A long-standing question for pollen tube growth is: what are the origins and biological identities of the apical vesicles that accumulated in the tip region of pollen tubes? Tip-focused exocytosis has traditionally attracted the most experimental attention and been suggested to play a central role in pollen tube growth guidance (Luo et al., 2017; Synek et al., 2017; Li et al., 2018). The *trans-*Golgi network (TGN) is regarded as an independent organelle serving as a hub for connecting multiple endomembrane sorting and trafficking pathways in plant cells. It is responsible for receiving, sorting, packaging and secretion of different types of cargoes to their target destinations for the proper functions (Lam et al., 2007; Richter et al., 2009; Uemura, 2016; Elliott et al., 2020). In the growing pollen tube tip, exocytic vesicles from TGNs are believed to be the main source and play crucial roles in cell polarization and growth (Wang et al., 2010; Stephan et al., 2014; Grebnev et al., 2020). For instance, Jia et al. (2018) recently demonstrated that disruption of TGN biogenesis and functional organization by mutation of a Golgi-localized protein loss of TGN (LOT) which is a component of the guanine nucleotide exchange factor (GEF) complex of small Rab GTPase Ypt6, significantly inhibited pollen tube tip growth by impairing pectic cell wall formation and apical localization of kinases and phosphoinositide. Thus, TGN and TGN-derived secretion vesicles are critical for pollen tube growth. However, are all of the exocytic vesicles concentrated in pollen tube tip solely derived from TGN? A recent study of *Nicotiana tabacum* pollen specific pectin methylesterase 1 (NtPPME1), a key pectin modification enzyme regulating the rigidity of cell wall (Bosch et al., 2005; Wang et al., 2013, 2016), reveals that the polar exocytosis and apical targeting of NtPPME1 are directly mediated Golgi-derived secretion vesicles (GDSVs) which by-pass the TGN in growing pollen tubes. Additionally, the fluorescent signal intensity and apical targeting of NtPPME1-GFP are closely associated with the growth oscillation and polarity switch during the pollen tube growth as illustrated in Figure 2A (Wang et al., 2013, 2016). Therefore, GDSV is believed to be an alternative type of apical exocytic vesicle governing the polar growth of pollen tubes. It further suggests that TGN-independent secretion vesicles could serve as an essential population of apical exocytic vesicles for supporting the pollen tube tip growth. On the other hand, TGN-mediated protein sorting and secretion, especially by clathrin coated vesicles (CCVs) which are facilitated by adaptor protein 1 (AP1), have been often considered as the major secretory pathway in plant cells (Wang et al., 2014). Therefore, one might speculate that whether the TGN-derived CCVs are likely to present in the tip region (Wang et al., 2014; Grebnev et al., 2020). Nevertheless, this scenario is not supported by the ultrastructural results of the apical vesicles that usually are non-coated (Ketelaar et al., 2008; Wang et al., 2010). Whether the apical exocytic vesicles from TGN are de-coated CCVs or different types of uncoated secretion vesicles still remain to be further explored. In addition, Prado and colleagues identified nanovesicles also named as pollensomes which are secreted from olive pollens during pollen germination and pollen tube growth are essential for plant fertilization. Further employment of vesicle isolation by sucrose gradient and Fourier transform infrared (FTIR) analysis of the pollensome identified that it is composed by a heterogeneous population of secretory vesicles carrying a diverse range of proteins including PME, olive pollen allergens, fructokinase, cytoskeletonal proteins and so forth to work together to sustain the apical pollen tube growth. Nevertheless, how the pollensome is involved in the pollen tube exocytosis and regulates the pollen tube growth remain to be further explored (Prado et al., 2014). Cumulatively, further elucidation of the molecular identities of different exocytosis vesicles and characterization of their biological functions will be necessary for understanding how the exocytosis drives and fine-tunes pollen tube growth and polarization.

In contrast to exocytosis, the working machinery and functions of endocytosis in growing pollen tubes is less understood. Early studies by employing FM dyes up-taken in growing pollen tubes have well demonstrated that rapid and vigorous endocytosis takes place at the tip and heavily stain the inverted cone region (Figure 2B), whereas slow rate of endocytosis also occurs in the pollen tube shank (Zonia and Munnik, 2008; Onelli and Moscatelli, 2013). Moreover, Moscatelli and colleagues found two distinct types of endocytosis: clathrin-mediated endocytosis (CME) and clathrin independent endocytosis (CIE) participate in tobacco pollen tube growth by uptaking and tracking of positive and negative charged nanogold particles. CME occurs at the tip and subapical regions, whereas CIE mediates vesicle trafficking to vacuoles (Moscatelli et al., 2007). CME is a conserved cellular process that couples sequential protein recruitment and lipid modifications with dramatic shape transformations of the plasma membrane in mammals, yeasts and plants. Due to the existence of cell wall, the turgor pressure in plant cells is higher than that of mammalian cells in order to push the PM firmly against the cell wall. It even reaches to ∼0.2 MPa in lily pollen tubes in order to drive the fast cell expansion (Liu et al., 2017). Therefore, the force and time needed for membrane inward invagination and vesicle pinching off during CME are likely to be stronger and longer in plant cells than that in mammals and yeasts. Although clathrin light chains (CLC) and heavy chains (CHC) as well as CME adaptor proteins are found localized in the apex, sub-apical and shank regions of pollen tubes (Blackbourn and Jackson, 1996; Kaneda et al., 2019), it seems that CME may be too slow to internalize membrane and proteins from the apical surface and maintain the cone shape of the pollen tube tip. Nevertheless, one should note that pollen tube growth oscillates and it can be separated into an active growing phase in which fast tip expansion occurs, and a resting phase where pollen tube growth is extremely slow or arrested (Damineli et al., 2017). The distribution of clathrin and CME adaptor proteins such as Arabidopsis epsin-like clathrin adaptor protein 2 (AtECA2) only localized to the subapical and shank region during the active growing state of the pollen tube. In contrast, they appear in the pollen tube apex during the resting phase of pollen tubes (Kaneda et al., 2019). These results are consistent with previous ultrastructural studies that CCVs have seldom been observed in the pollen tube tip (Ketelaar et al., 2008; Wang et al., 2010). Thus, CME is unlikely to be the major type of the endocytosis in the tip of growing pollen tubes.

What is then the identity of the endocytic vesicles in the growing pollen tube tip? Since the kinetics of CME is too slow, it is likely that a faster apical endocytic mechanism is employed to meet the needs. Actually, several alternative endocytic pathways termed as fast and ultrafast endocytosis which all belong to CIE have been found to rapidly remove receptors and proteins from the cell surface in reaction to stress hormones, membrane flux during directed cell migration and compensatory endocytosis after exocytosis of synaptic vesicles in animal cells (Onelli and Moscatelli, 2013; Watanabe and Boucrot, 2017). Although current studies on fast and ultrafast endocytosis have shown that they are not all constitutively active and may use different working mechanisms for rapid removal of receptors from cell surface, it will be worthwhile to figure out and characterize whether similar fast/ultrafast endocytosis occurs in the growing pollen tube tip. Further identification of specific molecular markers for apical exocytosis and endocytosis, respectively, will be crucial for understanding how the tip growth dome of pollen tubes is generated and maintained (Onelli and Moscatelli, 2013; Grebnev et al., 2017; Guo and Yang, 2020).

## The Sites for Exocytosis and Endocytosis in the Growing Pollen Tube Tip

In addition to the uncertain nature of the clear zone vesicles, another unsolved question of membrane dynamics at the pollen tube tip is: where are the exact sites for exocytic- and endocytic-vesicle fusion with the apical PM, respectively? The conventional model based on the tracking, distribution and quantitative analysis of FM dyes uptake in tobacco growing pollen tubes demonstrates that the endocytosis occurs in the pollen tube apex while exocytosis takes place in the sub-apical areas adjacent to the apex (Zonia, 2010; Zonia and Munnik, 2011; Grebnev et al., 2017). However, the conventional model is under debate because: (i) the tiny size of apical vesicles is beyond the limit of resolution of light microscopes and are therefore difficult to track; (ii) it is also technical challenging to use a 3D confocal laser scanning microscope to trace and distinguish the apical vesicles since they are very dynamic and overlapped in growing pollen tube tips (Grebnev et al., 2017).

Recently, several independent studies using fluorescence recovery after photobleaching (FRAP) of different types of exocytic proteins has demonstrated that exocytosis takes place in the apex region, the same region as for endocytosis (Lee et al., 2008; Luo et al., 2016; Wang et al., 2016; Campanale et al., 2017; Li et al., 2018; Grebnev et al., 2020). For example, NtPPME1, a pectic cell wall modification enzyme, is exocytosed and deposited in the apoplast of the pollen tube tip to regulate the rigidity of pollen tube tip cell wall. Expression of the chimeric fusion of NtPPME1 with GFP in growing tobacco pollen tubes reveals that NtPPME1 polarly localized to the pollen tube tip as illustrated in Figure 2A (Bosch et al., 2005; Wang et al., 2013, 2016). Application of FRAP demonstrates that the recovery of the fluorescent signal was firstly observed in the center of the apex, thereafter it gradually expanded to the flanks of apex (Wang et al., 2016). Consistently, Arabidopsis receptor-like kinase 1 (AtPRK1), a PM localized receptor, reaches the PM of pollen tubes *via* the exocytic pathway. The tip-localized AtPRK1-GFP was selectively bleached and the recovery of AtPRK1 also firstly started from the middle of apex and spread distally (Lee et al., 2008; Grebnev et al., 2020). It seems that pollen tube apex is a hot zone where both vigorous exocytosis and endocytosis take place simultaneously, although the underlying details of how the hot zone hosts and coordinates the spatiotemporal dynamics of exocytosis and endocytosis remain largely unexplored (Figure 1). Actually, the hot zone is an area equivalent to the principle site for clustering large amounts of synaptic vesicles needed for release, fusion and perhaps in vesicle retrieval after fusion in synapses. It is a region that clusters synaptic vesicles to increase the proximity between molecules on the synaptic vesicle membrane and the PM (Maritzen and Haucke, 2018). Despite the biological functions and the underpinning mechanism regulating the active zone in synapses and pollen tubes are likely to be different, the concept of an active zone can be introduced to describe the pollen tube apex where exocytic and endocytic vesicles are actively recruited and regulated as demonstrated Figure 1.

## Mathematical Modeling of Exocytosis and Endocytosis During Pollen Tube Polarization and Tip Growth

The generation of theoretical models by mathematical analysis has been employed as a useful tool in biological studies to better understand the molecular basis of cells. More importantly, it helps to develop a testable hypothesis, apply the modeling on broader similar biological systems and stimulate new experiments (Marco et al., 2007; Altschuler et al., 2008; Brannmark et al., 2017; Luo et al., 2017; Tian et al., 2019).

Pollen tube growth is guided by the signal cues released from the female gamete to make the switch of cell polarity to eventually allow the pollen tube to reach the ovule for fertilization (Wang et al., 2013; Zhong and Qu, 2019). The plant specific RhoGTPase 1 (ROP1) has been well demonstrated as a master regulator in pollen tube exocytosis and polarization. How does exocytosis, at the front line of pollen tube growth, function in the directional switch of pollen tube growth? Luo and colleagues developed a computational model to connect the tip-focused exocytosis with pollen tube growth guidance *via* ROP1. The model is firstly generated basing on the experimental estimation of the rate of GFP-ROP1 diffusion on the PM of Arabidopsis pollen tubes determined by FRAP and the strength of positive and negative feedback loops (*k*_pf_ and *k*_nf_) regulating active ROP1. Thereafter, the mathematical model is validated by comparing the parameters between the stimulated model prediction and actual experiments which examined the apical cell wall formation, pectin distributions and tip morphological shapes of the pollen tubes of the wild type and genetic mutants altered in ROP1 activation. After the proof of the model, they sought to explore model-inspired new insights into the roles of exocytosis in pollen tube growth guidance. The model then is employed to reproduce the connection between pollen tube polarity switch during growth guidance and exocytic parameters of the tip growth. The stimulating results from the modeling reveal a central role of exocytosis in coordinating pollen tube tip growth and guidance (Luo et al., 2017; Tian et al., 2019). Nevertheless, the model is only a simplified framework without considering endocytosis, calcium, pH, phosphoinositides and so forth which are also critical for pollen tube growth and guidance. Especially, it will be important to investigate how endocytosis participates in the tip expansion. Whether endocytosis is also centered in the apical signaling network to work together with exocytosis to dominate the pollen tube growth and guidance? Actually, live-cell tracing of the dynamics of endocytosis stained by FM4-64 has shown that apical endocytosis oscillates along with pollen tube growth rates and re-orientates together with cell polarity alteration as shown in Figure 2B (Wang et al., 2013). It indicates that endocytosis is also involved during pollen tube polarization and growth. However, the underpinning positive and negative regulatory network for endocytosis in pollen tube tip growth and guidance still remains obscure. Future studies are necessary toward elucidating how ROP1 regulates endocytosis in the pollen tube tip and what specific mechanisms are employed, respectively, to regulate exocytosis and endocytosis within the hot zone of pollen tube tip.

Indeed, endocytosis has been shown to be necessary for the dynamic maintenance of polarized membrane proteins in other model organisms and systems (Polo and Di Fiore, 2006). For example, studies in budding yeasts have shown that endocytosis optimizes the dynamic localization of membrane proteins which regulate cortical polarity. Furthermore, Marco et al. (2007) constructed a mathematical model and live-cell measured all the modeling parameters including dynamics of redistribution of polarized membrane proteins, cell cap morphology, membrane fluctuations, endocytosis rates, maintenance of cortical polarity in yeasts by expressing the activated Cdc42 which is a key Rho GTPase in regulating cell polarization. The results from the modeling simulation and experiments suggest that endocytosis mainly defines the spatial precision and morphology of the polarized state of membrane proteins in yeasts (Polo and Di Fiore, 2006; Marco et al., 2007; Howell et al., 2012). Based on the hints from the existing exocytosis models for pollen tube tip growth and endocytosis models in yeasts, it will be of great interest to generate a more comprehensive mathematical model by integrating exocytosis, endocytosis and pollen tube polar growth to investigate the working mechanism of how the polar growth of pollen tubes is dynamically achieved, maintained and regulated.

Besides, in the past decade, PM-localized receptors for sensing the guidance cues and maintaining the integrality of pollen tubes have been identified such as the Leucine-rich Repeat Extension (LRX) family proteins and *Catharanthus roseus* RLK1-like kinases (CrRLK1Ls) like Buddha’s Paper Seal 1/2 (BUPS1/2) and ANXUR1/2 (Ge et al., 2017; Mecchia et al., 2017; Peng et al., 2018). However, exactly how these receptors are exocytosed to the pollen tube PM and maintain their tip localization remains unexplored. Therefore, it will be essential in the future to test the established models from other cell systems with respect to growing pollen tubes to investigate whether apical endocytosis functions in optimization and maintenance of membrane proteins localized to the pollen tube tip.

## Perspectives

As our understanding comes into focus, tip-focused exocytosis and endocytosis appears as two sides of the same coin. Meanwhile, they coexist and reach a dynamic yin-yang balance which is not a fifty-to-fifty static state, but rather turning out as an anterograde growing equilibrium as shown in Figures 2C–E during pollen tube growth oscillation and guidance. It raises several interesting questions which are of worth to be tackled in the future: (i) What is the mechanism recruiting exocytosis and endocytosis coincidently in the pollen tube apex and counterbalancing them for the tip growth? (ii) Is there an intertwined feedback mechanism between exocytosis and endocytosis to balance these two directionally opposite trafficking processes for pollen tube tip polarization and growth? (iii) Whether and how apical endocytosis contributes to the dynamic distribution of tip-localized proteins and maintains their polarized localization? (iv) How does endocytosis participate in pollen tube growth guidance of signal sensing or response during plant fertilization? The experimental results and simulation models from mammalian and yeast systems may provide useful hints for the answers to the questions above. However, it is noteworthy that the unique features of the pollen tube makes it also different from other model cell types. Future studies by a combination of newly emerged advanced imaging approaches such as lattice light-sheet microscopy and 3D tomography together with genetic, biochemical as well as mathematical modeling will be useful to unravel the mysteries of pollen tube polarization, growth and guidance.

## Author Contributions

LZ, MR, and HW designed the concept and organized the manuscript. MR and HW wrote the first draft of the manuscript. LZ and HW revised and finalized the manuscript. All authors contributed to the article and approved the submitted version.

## Conflict of Interest

The authors declare that the research was conducted in the absence of any commercial or financial relationships that could be construed as a potential conflict of interest.
