# Erratum: Exocytosis and Endocytosis: Yin-Yang Crosstalk for Sculpting a Dynamic Growing Pollen Tube Tip

**DOI:** 10.3389/fpls.2020.638706

**Published:** 2021-01-06

**Authors:** Frontiers Production Office

**Affiliations:** Frontiers Media SA, Lausanne, Switzerland

**Keywords:** endocytosis, exocytosis, pollen tube growth, cell polarity, mathematical modeling

Due to a production error the affiliation “College of Life Sciences, South China Agricultural University, Guangzhou, China,” was incorrectly typeset as “College of Life Science, South China Agricultural University, Guangzhou, China.”

The publisher apologizes for this mistake. The original article has been updated.

